# Financing Long-Term Services and Supports: Opportunities for Overcoming Historical Roadblocks to Reform

**DOI:** 10.1093/geroni/igaa057.2529

**Published:** 2020-12-16

**Authors:** Edward Miller, Pamela Nadash, Marc Cohen

**Affiliations:** University of Massachusetts Boston, Boston, Massachusetts, United States

## Abstract

This presentation documents the continuing failure to tackle the problem of financing long-term services and supports (LTSS)—a failure most recently seen in the only national legislation ever enacted to comprehensively address LTSS costs: the Community Living Assistance Services and Supports (CLASS) Act. The CLASS Act was included in the Affordable Care Act, but was repealed in 2013. Subsequently, policy experts and some Democrats have made proposals for addressing the LTSS financing crisis. Moreover, significant government action is taking place at the state level, both to relieve financial and emotional burdens on LTSS recipients and their families and to ease pressure on state budgets. Lessons from these initiatives could serve as opportunities for learning how to overcome roadblocks to successful policy development, adoption, and implementation across states and for traversing the policy and political tradeoffs should a policy window open once again for addressing the problem of LTSS financing nationally.

